# Extraction, Structural Characterization, and In Vivo Anti-Inflammatory Effect of Alginate from *Cystoseira crinita* (Desf.) Borry Harvested in the Bulgarian Black Sea

**DOI:** 10.3390/md21040245

**Published:** 2023-04-16

**Authors:** Vesela Kokova, Paolina Lukova, Alexandra Baldzhieva, Plamen Katsarov, Cédric Delattre, Roland Molinié, Emmanuel Petit, Redouan Elboutachfaiti, Marianna Murdjeva, Elisaveta Apostolova

**Affiliations:** 1 Department of Pharmacology, Toxicology, and Pharmacotherapy, Faculty of Pharmacy, Medical University-Plovdiv, Vasil Aprilov Str. 15A, 4002 Plovdiv, Bulgaria; 2 Department of Pharmacognosy and Pharmaceutical Chemistry, Faculty of Pharmacy, Medical University-Plovdiv, 4002 Plovdiv, Bulgaria; 3 Department of Microbiology and Immunology, Faculty of Pharmacy, Medical University-Plovdiv, Vasil Aprilov Str. 15A, 4002 Plovdiv, Bulgaria; 4 Research Institute at Medical University-Plovdiv, Vasil Aprilov Str. 15A, 4002 Plovdiv, Bulgaria; 5 Department of Pharmaceutical Sciences, Faculty of Pharmacy, Medical University-Plovdiv, Vasil Aprilov Str. 15A, 4002 Plovdiv, Bulgaria; 6 Clermont Auvergne INP, CNRS, Institut Pascal, Université Clermont Auvergne, 63000 Clermont-Ferrand, France; 7 Institut Universitaire de France (IUF), 1 Rue Descartes, 75005 Paris, France; 8 UMRT INRAE 1158 BioEcoAgro, BIOlogie des Plantes et Innovation (BIOPI), Avenue des Facultés, IUT d’Amiens, Université de Picardie Jules Verne, Le Bailly, 80025 Amiens, France

**Keywords:** alginate, *Cystoseira crinita*, anti-inflammatory effect, rat paw edema, histamine, cytokines, IL-1β, TNF-α, Il-6, peritonitis

## Abstract

The aim of this study was to identify the chemical composition and sequential structure of alginate isolated from *C. crinita* harvested in the Bulgarian Black Sea, as well as its effects in histamine-induced paw inflammation in rats. The serum levels of TNF-α, IL-1β, IL-6, and IL-10 in rats with systemic inflammation, and the levels of TNF-α in a model of acute peritonitis in rats were also investigated. The structural characterization of the polysaccharide was obtained by FTIR, SEC-MALS, and ^1^H NMR. The extracted alginate had an M/G ratio of 1.018, a molecular weight of 7.31 × 10^4^ g/mol, and a polydispersity index of 1.38. *C. crinita* alginate in doses of 25 and 100 mg/kg showed well-defined anti-inflammatory activity in the model of paw edema. A significant decrease in serum levels of IL-1β was observed only in animals treated with *C. crinita* alginate in a dose of 25 mg/kg bw. The concentrations of TNF-α and IL-6 in serum were significantly reduced in rats treated with both doses of the polysaccharide, but no statistical significance was observed in the levels of the anti-inflammatory cytokine IL-10. A single dose of alginate did not significantly alter the levels of the pro-inflammatory cytokine TNF-α in the peritoneal fluid of rats with a model of peritonitis.

## 1. Introduction

The current tendency towards the application of natural products in medicine focuses on marine organisms and their unique molecules. Polysaccharides from macroalgae have demonstrated different pharmacological effects, including antimicrobial, antiviral, anticancer, anticoagulant, antimutagenic, hypolipidemic, hypoglycaemic, antioxidant, immunomodulating, anti-inflammatory, antinociceptive, angiogenic, and gastro- and cardioprotective bioactivities [1,2,3,4]. Attempts to establish a relationship between the structures of the polysaccharides and their bioactivities are a challenge due to the complexity of these polymers. Most of the polysaccharides are highly branched heteropolymers with different substituents groups on the various carbon atoms of their main chain and side-sugar components. Furthermore, the monosaccharide composition and the glycosidic bonds between monosaccharides can be very heterogeneous. Additionally, this heterogeneity also depends strongly on the species of algae from which they are extracted, as well as the period of harvesting and the geographical location of the collection site [4].

The marine macroalgae belong to three main phyla: *Chlorophyta* (green algae), *Rhodophyta* (red algae), and *Phaeophyta* (brown algae). In recent years, brown algae have been widely used as a source of biologically active substances with diverse applications in the medicine, pharmacy, and food industries. Three main groups of polysaccharides have been isolated from brown algae: sulfated polysaccharides (fucoidans), acidic polysaccharides (alginic acid and alginates), and glucans (laminarin) [5].

Fucoidans are obtained from the cell walls and intercellular spaces of the genera *Fucus*, *Cystoseira*, *Ascophyllum*, *Sargassum*, *Padina*, etc. [6]. Generally, the chemical composition of fucoidans is very complex; nevertheless, their structural backbone is composed of repeating α-(1,3) linked L-fucopyranose residues or alternating α-(1,3) and α-(1,4) linked L-fucopyranoses. Along with the fucosyl main chain, a wide range of other monosaccharides (mannose, galactose, arabinose, xylose, and glucose), uronic acids, and proteins may also be part of the fucoidan structure. Fucoidans have been studied intensively during the last few years regarding their multiple biological activities as antitumor, immunomodulatory, antiviral, antimicrobial, antidiabetic, nephroprotective, anti-inflammatory, and anticoagulant agents [7].

Laminarin was found in higher amounts in the cell vacuoles of brown algae of the genera *Laminaria* and *Saccharina* [8]. It is a highly water-soluble, branched polysaccharide made of a linear β-(1,3)-linked glucose-based chain with β-(1,6) branching of mannitol or glucose residues. Laminarin contributes to dietary fiber intake and has antibacterial, antitumor, antioxidative, and anticoagulant properties [9].

Alginates are found abundantly in the cell walls of the genera *Padina*, *Turbinaria*, *Sargassum*, *Cystoseira*, *Dictyota*, *Fucus*, *Hydroclathrus*, etc. [10]. Alginate can be considered as a chain-forming heteropolysaccharide composed of *β*-(1–4)-linked D-mannuronic (M) and *α*-(1,4)-linked L-guluronic (G) acids, arranged either as homopolymeric (MM or GG) or heteropolymeric (MG or GM) blocks [11]. The composition of the blocks is a specific feature of alginate polysaccharides and can also be influenced by the parameters mentioned above, namely, the species used for the extraction, the part of the thallus from which the extraction is made, the locality of harvest, and the harvest time [1].

Alginate is applied in a wide variety of industrial processes, such as food and textile manufacturing, as well as in the pharmaceutical industry and biotechnology for microencapsulation, drug delivery, and tissue engineering [12,13]. Moreover, various biological activities of alginate and its derivatives, including antitumor, antioxidant, immunoregulatory, anti-inflammatory, neuroprotective, mucoprotective, antibacterial, hypolipidemic, antihypertensive, hypoglycemic, prebiotic, suppression of obesity, and promotion of cell proliferation, have been described [14,15].

*Cystoseira crinita (C. crinita)* is a brown macroalga that is widespread on the coastal line of the Mediterranean Sea and in the Black Sea. Seven sterols and volatile compounds, mainly terpenoids, have been determined in this species [16]. Our recent study has determined the chemical composition and structure characteristics of fucoidan isolated from *C. crinita.* Furthermore, we have reported that this sulfated polysaccharide exhibited a well-defined anti-inflammatory activity [7]. No data have been found in the literature about the anti-inflammatory effect of alginate isolated from *C. crinita*.

The first aim of this study was to determine the chemical composition and structure characteristics of alginate isolated from *C. crinita* harvested in the Bulgarian Black Sea. Our next aim was to evaluate its effects in a histamine-induced model of inflammation in rats, and on the serum levels of tumor necrosis factor-α (TNF-α), interleukin-1 β (IL-1β), interleukin-6 (IL-6), and interleukin 10 (IL-10) in rats with lipopolysaccharide-induced systemic inflammation. The levels of TNF-α in a model of acute carrageenan-induced peritonitis in rats were also investigated.

## 2. Results

### 2.1. Extraction Yield and Chemical Composition of C. crinita Alginate

The results of the extraction yield and colorimetric analyses of *C. crinita* alginate are summarized in Table 1. The extraction yield was quantified as 20.18 ± 1.72% calculated on algae dry weight. The obtained *C. crinita* alginate consisted mainly of uronic acids (50.14%) and minor amounts of neutral sugars (19.66%), which was in accordance with the literature data for alginates extracted from other brown algae species [17,18]. The turbidimetric assay revealed a low amount of sulfate groups (0.63%), indicating that the possibility of contamination of alginate polysaccharides by fucoidan residues was negligible. Protein concentrations (<0.04%) and the total polyphenols (<0.10) were present only in trace amounts due to the purification procedures on the algae dry mass [7].

### 2.2. FTIR Spectroscopy Analysis

**Figure 1:**
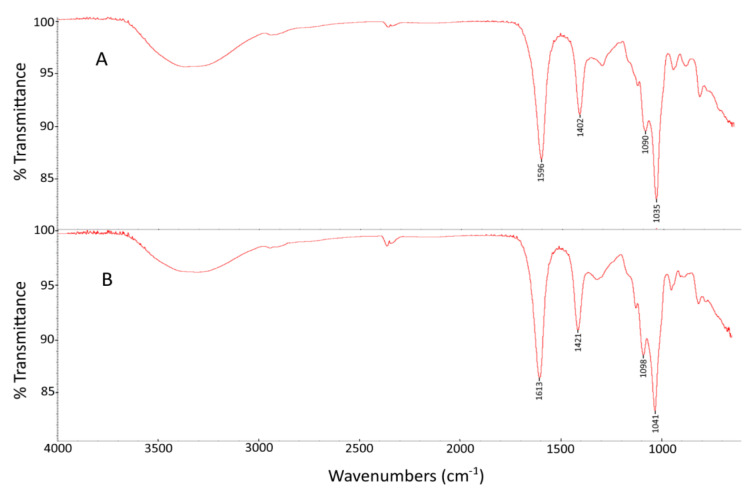
FTIR of (**A**) alginate extracted from *C. crinita* and (**B**) alginate standard from Sigma Aldrich (Inc. St. Louis, MO, USA).

*C. crinita* alginate was analyzed by Fourier-transform infrared (FTIR) spectroscopy (Figure 1A) and the obtained signals were analogous to the infrared spectra of the standard alginate purchased from Sigma-Aldrich (Figure 1B). The peaks observed at 3328 cm^−1^ and 2953 cm^−1^ were attributed to O–H and C–H stretching vibrations, respectively [18]. The band at 1596 cm^−1^ was attributed to the asymmetric C–O stretching vibrations, while the band at 1402 cm^−1^ was associated with the symmetric C–O vibrations [19]. The absorption peaks at 1090 and 1035 cm^−1^ were characteristic signals for the alginate polysaccharide and were attributed to the mannuronic and guluronic units, respectively [17]. Moreover, Pereira et al. found that the ratio of these two absorption band intensities could be used for the estimation of the M/G ratio [20]. The FTIR assay revealed the absence of signals around 1230–1280 cm^−1^, related to the sulfate ester groups (S=O), which confirmed the purity of the extracted alginate [21].

### 2.3. Proton Nuclear Magnetic Resonance (^1^H NMR) Spectroscopy

**Figure 2:**
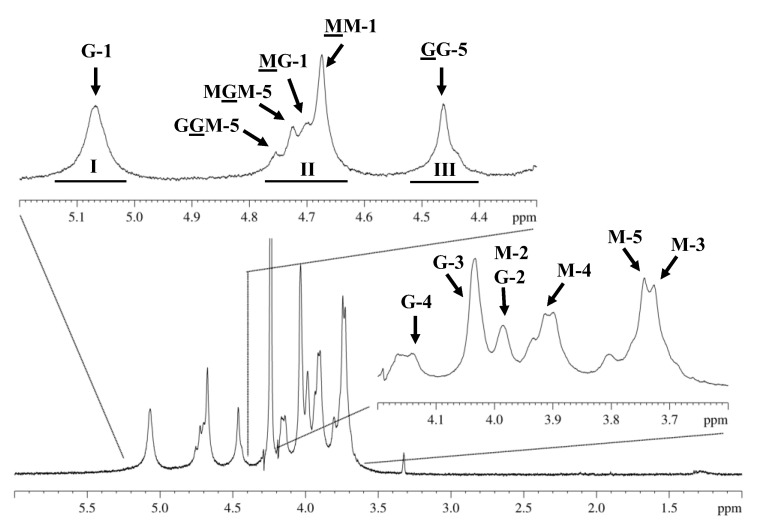
Expanded regions of the ^1^H NMR spectrum (80 °C) of sodium alginate extracted from *C. crinita* harvested in the Bulgarian Black Sea. M and G refer to protons of D-mannuronic acid and L-guluronic acid residue, respectively. Signal I = guluronic acid anomeric proton (G-1), signal II = overlap between the mannuronic acid anomeric proton (M-1) and the H-5 of alternating blocks (GM-5), signal III = guluronic acid H-5 position (block GG-5). Underlined M and G denote signals from M and G residues, respectively, whereas letters not underlined denote neighboring residues in the polymer chain. Numbers indicate which proton is causing the signal.

The complete characterization of chemical composition and block distribution of alginate molecules was explored by using a consistent methodology via proton nuclear magnetic resonance (^1^H NMR) spectroscopy [22]. The results obtained for the alginate extracted from the studied *C. crinita* algae showed typical 500 MHz-^1^H NMR data (Figure 2) with a set of signal characteristics assigned to guluronic and mannuronic acid repeating units [23,24]. The signal assignment was achieved by the comparison with previously reported chemical shifts [25,26]. The spectrum showed the five peaks (G-1, 5.07; G-2, 3.98; G-3, 4.03; G-4, 4.14; and G5, 4.46) corresponding to L-guluronic acid and another five peaks (M-1, 4.67; M-2, 3.98; M-3, 3.72; M-4, 3.91; and M-5, 3.74) typical of D-mannuronic acid. The chemical shifts are in agreement with those reported in the literature.

The expanded regions between 4.3 and 5.2 ppm also showed, in addition to the signals attributed to the anomeric hydrogen of guluronic acid (G-1) at 5.0–5.15 ppm (A_I_) and the H-5 of guluronic acid residues in the homopolymeric (GG-5) blocks at 4.4–4.5 ppm (A_III_), the anomeric hydrogens of mannuronic acid (M-1) overlapped with the H-5 of alternating blocks (GM-5) at 4.6–4.8 ppm (A_II_).

Likewise, the ^1^H NMR spectra of the analyzed alginate from *C. crinita* did not reveal any traces of fucoidans in the sample, identified by the characteristic signal of the methyl group in fucose ~1.3 ppm. This result is in agreement with that obtained with the turbidimetric assay, where most of the sulfate groups were situated on the fucose units of fucoidan polysaccharides.

The area of described signals (A_I_, A_II_, and A_III_) was calculated and used to determine the M/G ratio, the molar pair frequencies of the monads of guluronic acid and mannuronic acid (F_G_ and F_M_), and the diad sequences (F_GG_, F_MM_, F_MG_ or F_GM_). The proportions of each block (F_G_ and F_M_), the homogeneous (F_GG_ and F_MM_), and heterogeneous (F_GM_ and F_MG_) blocks of alginate extracted from *C. crinita* were estimated by applying the commonly used equations of Grasdalen et al. [25], summarized as follows:

F_G_ = A_I_/(A_II_ + A_III_) (1)

F_M_ = 1 − F_G_ (2)

F_GG_ = A_III_/(A_II_ + A_III_) (3)

F_GM_ = F_G_ − F_GG_ (4)

F_MM_ = F_M_ − F_GM_ (5)

M/G = F_M_/F_G_ (6)

Different details on the chemical composition of the alginate extracted from *C. crinita,* summarized in Table 2, are derived from integral ^1^HNMR values, along with the equations mentioned above.

### 2.4. SEC-MALS Analysis

**Figure 3:**
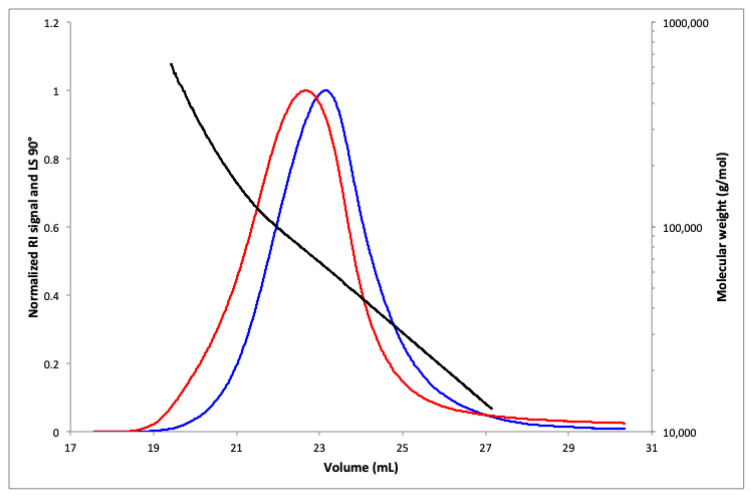
SEC-MALS chromatogram of *C. crinita* crude alginate giving Mw (g/mol) versus V (mL) (black), RI signal (blue), and light scattering at 90° (red).

The average molecular weight in mass (Mw) and average molecular weight in number (Mn) of alginate extracted from *C. crinita* were determined by SEC-MALS analysis. The elution profile is shown in Figure 3, demonstrating that the polymer has a homogeneous distribution. The calculated Mn and Mw were 5.29 × 10^4^ g/mol and 7.31 × 10^4^ g/mol, respectively. The narrow range of mass distribution given by the polydispersity index (PDI = 1.38) confirmed the homogeneity of the alginate polymer and indicated that the extraction process had a minimal impact on the degradation of the obtained polysaccharide [17,18]. The sample recovery rate was over 90%.

### 2.5. Effect of Alginate on Histamine-Induced Paw Edema

**Figure 4:**
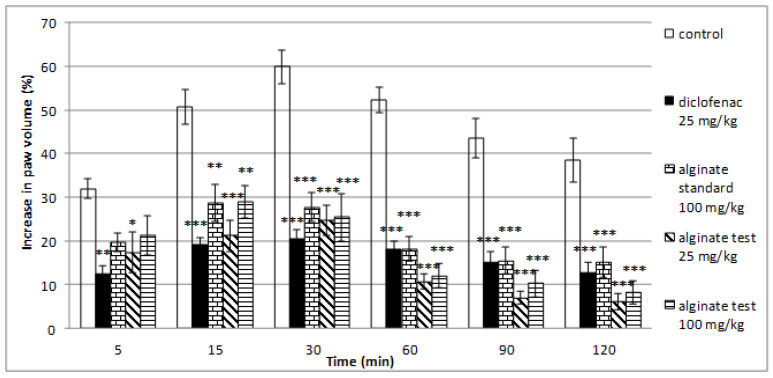
Effects of diclofenac, alginic acid sodium salt standard (100 mg/kg bw), and alginate test from *C. crinita* (25 and 100 mg/kg bw) on paw edema induced by histamine in rats. * *p* < 0.05 vs. controls at the same time; ** *p* < 0.01 vs. controls at the same time; *** *p* < 0.001 vs. controls at the same time.

Alginate isolated from *C. crinita* showed well-defined anti-inflammatory effects in the model of histamine-induced paw edema (Figure 4). The lower dose of alginate (25 mg/kg) caused a significant inhibition of the paw edema at all tested minutes: at the 5th min (17.39 ± 4.68 vs. 31.94 ± 2.23; *p* < 0.05), at the 15th min (21.23 ± 3.57 vs. 50.72 ± 4.05; *p* < 0.001), at the 30th min (24.64 ± 3.43 vs. 59.94 ± 3.85; *p* < 0.001), at the 60th min (10.73 ± 1.70 vs. 52.32 ± 2.98; *p* < 0.001), at the 90th min (7.04 ± 1.47 vs. 43.44 ± 4.52; *p* < 0.001), and at the 120th min of the experiment (6.10 ± 1.85 vs. 38.58 ± 5.03; *p* < 0.001) in comparison with the control group. A significant anti-inflammatory effect was also observed in the group treated with the higher dose of alginate (100 mg/kg) at the 15th min (28.99 ± 3.75 vs. 50.72 ± 4.05; *p* < 0.01), at the 30th min (25.46 ± 5.46 vs. 59.94 ± 3.85; *p* < 0.001), at the 60th min (12.16 ± 2.83 vs. 52.32 ± 2.98; *p* < 0.001), at the 90th min (10.37 ± 3.06 vs. 43.44 ± 4.52; *p* < 0.001), and at the 120th min (8.27 ± 2.53 vs. 38.58 ± 5.03; *p* < 0.001) after the histamine application when compared with controls. A significant antiphlogistic activity of alginate standard was registered at the 15th min (28.73 ± 4.26 vs. 50.72 ± 4.05; *p* < 0.01), at the 30th min (27.72 ± 3.34 vs. 59.94 ± 3.85; *p* < 0.001), at the 60th min (18.19 ± 2.79 vs. 52.32 ± 2.98; *p* < 0.001), at the 90th min (15.53 ± 3.01 vs. 43.44 ± 4.52; *p* < 0.001), and at the 120th min of the testing (15.23 ± 3.46 vs. 38.58 ± 5.03; *p* < 0.001) in comparison with controls. The isolated alginate from *C. crinita* showed more efficiency in the inhibition of paw edema when compared with the alginate standard during the late stages of inflammation (from 30th to 120th min; Figure 4, Table 3).

### 2.6. Effect of Alginate on the Levels of Pro-Inflammatory Cytokines (IL-1β, TNF-α, and IL-6) in Serum and Peritoneal Fluid

**Figure 5:**
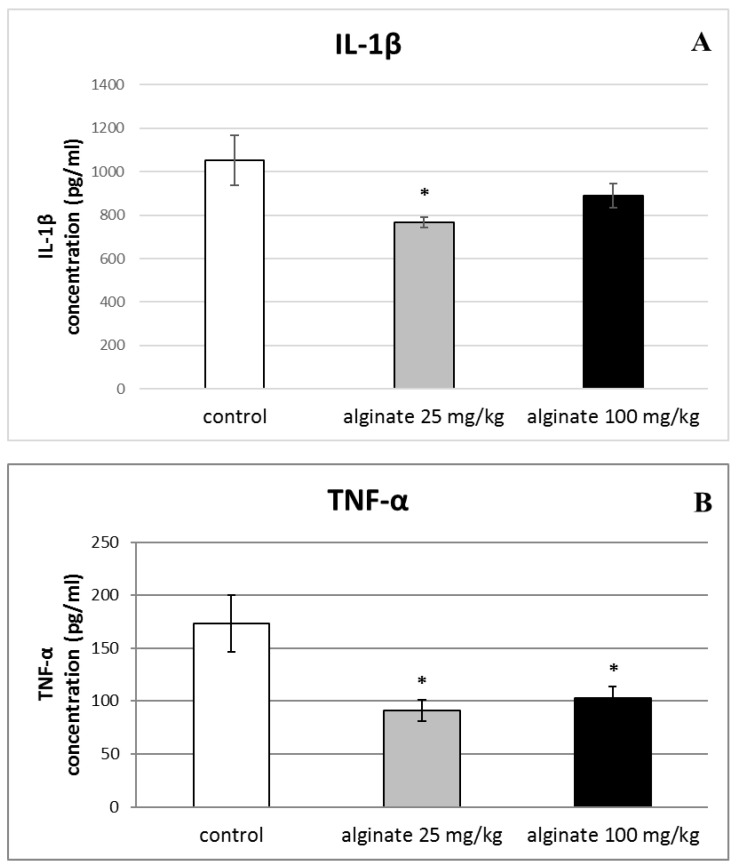

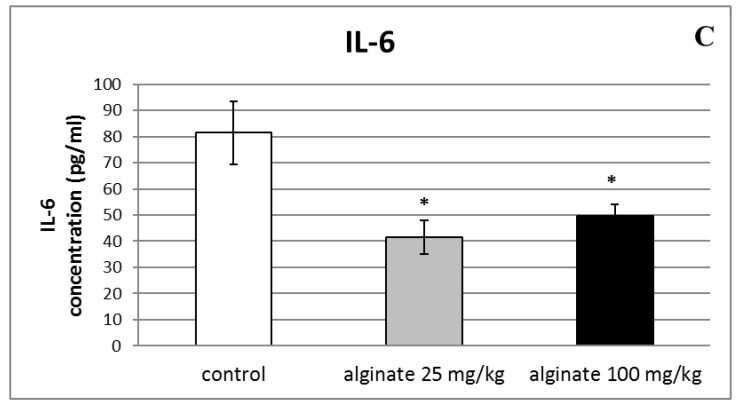
Effect of a single application of alginate from *C. crinita* (25 and 100 mg/kg bw) on serum levels of the pro-inflammatory cytokines IL-1β (panel **A**), TNF-α (panel **B**), and Il-6 (panel **C**) in LPS-induced systemic inflammation in rats. * *p* < 0.05 vs. same cytokine controls.

A significant decrease in serum levels of IL-1β was observed only in animals treated with the lower dose of alginate from *C. crinita* (25 mg/kg bw) in comparison with controls (766.07 ± 22.80 vs. 1052.58 ± 114.71; *p* < 0.05; Figure 5A). As shown in Figure 5B, the levels of TNF-α in the serum of rats treated with both doses of *C. crinita* alginate were significantly reduced when compared with the control group. Furthermore, the decrease was more distinguished in the group treated with the lower dose of alginate from *C. crinita* (25 mg/kg bw) than with the higher dose (100 mg/kg bw). The measured levels were 91.33 ± 10.09 vs. 173.48 ± 26.83 (*p* < 0.05) for the lower dose and 102.49 ± 11.61 vs. 173.48 ± 26.83 (*p* < 0.05) for the higher dose, respectively. The decreasing effect was also observed in IL-6 serum levels of rats treated with alginate isolated from *C. crinita* in a dose of 25 mg/kg bw (41.48 ± 6.50 vs. 81.46 ± 12.05; *p* < 0.05) and 100 mg/kg bw (49.54 ± 4.58 vs. 81.46 ± 12.05; *p* < 0.05) in comparison with controls (Figure 5C). Again, the effect was more prominent when the lower dose was applied.

**Figure 6:**
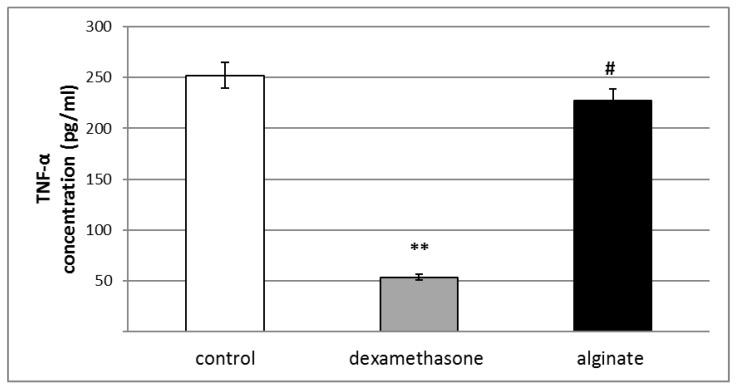
Effect of a single application of dexamethasone (0.2 mg/kg bw), and alginate from *C. crinita* (25 mg/kg bw) on the levels of TNF-α in the peritoneal fluid of rats with carrageenan-induced peritonitis. ** *p* < 0.01 vs. controls, # *p* < 0.05 vs. dexamethasone.

A single dose of alginate isolated from *C. crinita* did not significantly change levels of the pro-inflammatory cytokine TNF-α in the peritoneal fluid of rats with a model of peritonitis in comparison with controls (Figure 6).

### 2.7. Effect of Alginate on the Levels of Anti-Inflammatory Cytokine (IL-10) in Blood Serum

**Figure 7:**
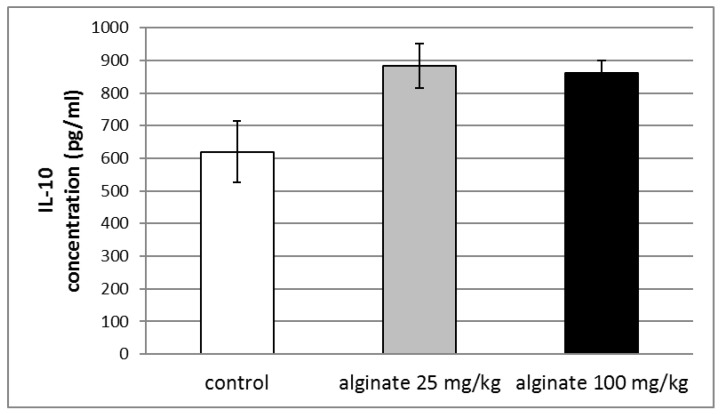
Effects of a single dose of alginate from *C. crinita* (25 and 100 mg/kg bw) on serum levels of IL-10 in rats with LPS-induced systemic inflammation.

The administration of single doses of alginate isolated from *C. crinita* showed a tendency to increase the serum levels of the anti-inflammatory cytokine IL-10 in rats with LPS-induced systemic inflammation, but no statistical significance was observed (Figure 7).

## 3. Discussion

For the first time, a structural characterization of alginate isolated from *C. crinita* harvested in the Bulgarian Black Sea coast was reported. The extraction yield of *C. crinita* alginate (20.18 ± 1.7%) was close to the values mentioned for *C. barbata* alginate (19%) harvested in the Romanian Black Sea coast [24] and Tunisian *C. compressa* alginate (21.65%) [18]. Moreover, the obtained yield was higher compared with other *Cystoseira* species, such as the Tunisian *C. barbata* (9.9%) [17] and *C. sedoides* alginate (11%) [18]. The alginate content of *C. crinita* algae determined in this study was similar to that found for other brown algae from different botanical genera such as *Dictyota* (*D. dichotoma* and *D. ciliolata* 20–21%), *Sargassum* (*S. fluitans* 21.1–22.8% and *S. oligocystum* 18.9–20.5%), *Padina* (*P. boergesenii* 24.3% and *P. gymnospora* 21.5%), *Turbinaria* (*T. triquetra* 22% and *T. ornata* 20.2%), [27,28,29,30]. Nevertheless, Bulgarian Black Sea alginate had a lower extraction yield compared with the species used for the industrial production of alginate, such as *Durvillaea willana* and *D. antarctica* (44–53%), *Macrocystis pyrifera* (43%), *Laminaria digitata* (40%), and *Ecklonia cava* (35–38%) [31,32,33].

The calculated M/G ratio of *C. crinita* alginate was 1.018, showing approximately equivalent amounts of both *β*-D-mannuronic (F_M_ = 0.505) and *α*-L-guluronic (F_G_ = 0.495) acids. Similar results were reported in other research works performed on different *Cystoseira* species. For example, alginates extracted from *C. myriophylloides* and *C. caespitosa* had a M/G ratio of 1.12 and 0.92, respectively [34,35]. These values can be considered as high since many algal alginates have M/G (mannuronic/guluronic) ratios within 0.45 and 1.85 [36]. Based on the results of the current literature, it appears that M/G ratios depend on the biological source and growth conditions, maturity, seasonal environment, and the extraction method [35]. In addition, the value of the M/G ratio also determines the biological effects of alginate polymers. Generally, a high M/G ratio corresponds to a higher biological activity [18,23]. The M/G obtained in this work indicates the potential of the alginate from *C. crinita* for the development of novel substances with a broad spectrum of biological properties.

Moreover, the various biological activities of alginate polysaccharides often correlate with their physical properties in aqueous medium. The physicochemical properties of alginate depend not only on the M/G ratio but also on the distribution of M and G units along the polymer chain [37]. Based on the data obtained in this study, the homopolymorphic MM or GG regions (F_MM_ = 0.289; F_GG_ = 0.280) were presented in a distribution format almost equivalent to the alternating blocks (F_MG_ and F_GM_, 0.216). These distributions were almost identical to those encountered in the alginate from *Fucus vesiculosus* (F_MM_ = 0.330; F_GG_ = 0.260; F_MG_ and F_GM_, 0.210) [11,38]. Likewise, the M/G ratio of the alginate extracted from *Fucus vesiculosus* was estimated as 1.17, with an F_G_ and F_M_ evaluated as 0.46 and 0.54 respectively. These structural data of alginate from *Fucus vesiculosus*, which is close to that of alginate from *C. crinita*, contribute strongly to the biological properties of these polysaccharides.

A complete description of the alginate monomer sequence is not possible only by the diads analysis (F_GG_, F_MM_, F_GM_, and F_MG_), as described previously [25]. Nevertheless, the η parameter, defined as η = F_MG_/(F_M_ × F_G_), can be used to predict the sequence distributions in algal alginates. Therefore, when η values < 1, this indicates the abundance of MM and GG homopolymeric block types, whereas η = 1 reveals completely random cases, and when 1 < η < 2, this illustrates the alternate-like cases of MG and GM. The η parameter obtained for the alginate extracted from *C. crinita* was estimated as 0.86 (η < 1). This is comparable to alginate from *Fucus vesiculosus (*η = 0.846 < 1) and suggests a polymer structure with a homopolymeric block distribution.

It is noteworthy that the biological properties of alginates are closely related to their structure. Several factors may influence their biological activity, including molecular weight, M and G content (M/G ratio), and spatial conformation of alginate molecules.

The estimated molecular weight is not very high but close to that of other Cystoseiraceae species. *C. compressa* alginate’s molecular weight has the most similarity to that analyzed in this study, with an estimated Mn and Mw in the range of 4.00 × 10^4^ to 6.50 × 10^4^ g/mol and 8.50 × 10^4^ to 1.00 × 10^5^, respectively [18,39]. Other species such as *C. barbata* have a slightly higher average molecular weight (1.26 × 10^5^ to 2.99 × 10^5^ g/mol) [17,24]. It has been demonstrated that, in *C. schiffneri* algae, the major factor influencing the Mw is the algae harvest period, with variations ranging from 4.49 × 10^3^ to 1.23 × 10^6^ g/mol. According to the authors, the significant seasonal variations of Mw are due to the life cycle of the algae. During the growth period, new stems containing low Mw polysaccharides are present. They continue to grow by gaining higher Mw alginates and, in the resting stages (December), a new thallus can be formed from old stems containing alginate polysaccharides with a high molecular weight [19].

The anti-inflammatory activities of alginates have been described previously. Most studies have shown biocompatibility and anti-inflammatory effects of alginates [40,41,42,43]. Sodium alginate possesses a local anti-inflammatory effect and is used for rectal administration in cases of chronic hemorrhoids and proctosigmoiditis, and chronic anal fissures after surgical intervention in the region of the rectum [44]. Sodium alginate is also reported to promote the regeneration of the mucous membranes, suppress inflammation in the stomach, and help the restoration of intestinal microbial flora [45]. Sodium alginate possesses a beneficial effect on the inflammation of gastric and esophageal mucosa, and radiation stomatitis [46]. Katayama et al. reported that poraprezinc–sodium alginate has a mucosal protective effect, an activity that is related to hemostatic properties, free radical scavenging, and tissue-repair-promoting action against hemorrhagic erosion and ulcers in patients with gingivostomatitis [47].

However, some research data show the pro-inflammatory potential of alginate. Yang & Jones (2009) reported that sodium alginate (molecular weight: 9500 kDa, M/G 1.96) induced RAW264.7 macrophages. It increased levels of the pro-inflammatory cytokines IL-1β, IL-6, IL-12, and TNF-α, and stimulated innate immune responses through NF-κB activation [48]. There is a report that high mannuronic-acid-containing alginate (HMA) from *Macrocystis pyrifera* has an immunostimulating effect and increases the number of peritoneal macrophages in mice. Furthermore, HMA induces phagocytosis, and the secretory and tumoricidal activity of macrophages, which is mediated by the production of cytokines and cytotoxic molecules (NO, H_2_O_2_, and TNF-α) [49]. Otterlei et al. (1991) showed that alginate with high M- and MG-blocks (isolated from *Ascophyllum nodosum*) were much more potent in inducing IL-1, IL-6, and TNF-α cytokine production compared with alginate with high G blocks (prepared from *Laminaria digitata*) [50]. Mannoglucan showed also TNF-α-like antitumor activity [51]. These results are in contrast with the anti-inflammatory activity of alginate demonstrated in our study. The possible explanation may be related to the fact that the experiments in these studies were performed in vitro, in contrast to our study, which was performed in vivo. On the other hand, the previous data show that the chemical composition of alginate may affect the immunogenicity of polymers. The predominance of mannuronic acid in the composition of alginate may be a factor that affects the immunogenicity of the polysaccharide and enhances its immunostimulating effect [49,50,51]. This hypothesis is supported by the difference in the M/G ratio. Yang & Jones’ (2009) experiments were performed with alginate with an M/G ratio of 1.96, while in our studies the content of mannuronic acid was lower (M/G ratio 1.02). It can be concluded that alginate with a high mannuronic acid content induces the synthesis of pro-inflammatory cytokines and stimulates the innate immune response by activating the NF-κB signaling pathway. The influence of other factors, such as molecular weight, algal source, extraction method, etc., should also be taken into account.

Similar to our results, other authors also reported an anti-inflammatory effect of alginate in vivo. Mirshafiey A and Rehm B.H. (2009) researched the effects of alginate gels after oral and intraperitoneal administration in experimental models of ulcerative colitis and glomerulonephritis, and the results showed that alginate can provide therapeutic efficacy in inflammatory diseases [52]. In a rat model of acute colitis, the oral administration in drinking water of low-viscosity sodium alginate (LVA), purified from *Macrocystis pyrifera* at a concentration of 0.5% (*w*/*v*), for one week inhibited the progression of colonic inflammatory lesions and reduced serum and colonic mucosal IL-6, TNF-α, leukotriene B4 (LTB4), and prostaglandin E2 (PGE2) levels [53]. In a rat model of chronic ulcerative colitis, LVA purified from *Macrocystis pyrifera* administered orally at a concentration of 0.5% (*w*/*v*) in drinking water for six weeks reduced the colonic damage score and serum levels of TNF-α, IL-6, LTB4, and PGE2, as well as colonic mucosal production of IL-6, TNF-α, and LTB4 [54]. In a rat model of immune complex glomerulonephritis, the application of LVA purified from *Macrocystis pyrifera* in a dose of 50 mg/kg bw i.p. showed a significant reduction in proteinuria and serum creatinine, and suppressed antibody production and the glomerular deposition of immune complex, as well as the development of glomerular lesions [55].

Free radicals are important mediators that cause or sustain inflammatory processes and, consequently, their neutralization by antioxidants and radical scavengers can attenuate inflammation [56]. There is a report that alginic acid isolated from brown algae *Sargassum wightii* in a dose of 100 mg/kg administrated orally exhibited anti-inflammatory and antioxidant effects in complete Freund’s-adjuvant-induced arthritis in rats. In this study, alginic acid decreased paw edema, the activities of COX-2 and 5-LOX, and neutrophil infiltration. Alginic acid reduced lipid peroxidation by modulating the cellular antioxidant defense system, and increasing the activity of antioxidant enzymes and the level of reduced glutathione [57]. Mo et al. (2003) found that alginate inhibited the TNF-α-induced expression of intercellular adhesion molecule-1, the production of nitric oxide, and H_2_O_2_ [58], revealing the possible role of the antioxidant properties of alginate in its anti-inflammatory activity.

Histamine is a chemical mediator with numerous effects: it stimulates gastric acid secretion, and plays a role in anaphylactic responses, inflammation, and neurotransmission. For investigating the early stage of the inflammatory response, the histamine-induced model of inflammation is often used. Our results showed that alginate isolated from *C. crinita* exhibited significant anti-inflammatory activity in histamine-induced paw edema in rats. An inhibitory effect of various types of alginic acid on histamine release from mast cells and hyaluronidase was also reported by Asada et al. (1997). Additionally, alginic acids with an M/G ratio of 1.0 and with higher molecular weight (150 to 370 kDa) exhibited the strongest inhibition of both activities. Free or charged carboxyl residues of uronic acids in alginate polymers appear to be essential for these effects because the esterification of the carboxyl residue in alginic acids resulted in enhancement of mast cell degranulation but without inhibition of the hyaluronidase activity [59]. In an allergy model, alginic acid from *Macrocystis pyrifera* composed of approximately 61% mannuronic and 39% guluronic acid, inhibited dose-dependently the systemic anaphylaxis and passive cutaneous anaphylaxis in experimental animals. It also decreased histamine release from serum and peritoneal mast cells, and histamine synthesis from its precursor L-histidine by inhibiting the enzyme histidine decarboxylase. All these effects were stronger than those of disodium cromoglycate used as a reference drug. Furthermore, alginic acid inhibited IL-1β and TNF-α secretion and mRNA expression but not IL-6 and IL-8 production in vitro [60].

The anti-phlogistic activity of alginate in histamine-induced paw edema in rats is probably related to a decreased secretion of pro-inflammatory cytokines. In our study, we observed reduced levels of the pro-inflammatory cytokines IL-1β, TNF-α, and IL-6 in rat serum after the application of alginate from *C. crinita*. In our experiments, the treatment with alginate from *C. crinita* did not significantly change the serum levels of anti-inflammatory cytokine IL-10. In rats with carrageenan-induced peritonitis, no significant changes in the levels of TNF-α were observed after a single dose of alginate isolated from *C. crinita*. Expression of inflammatory cytokines, including TNF-α, IL-1, and IL-6, is dependent on the activation of a transcription factor, nuclear factor (NF)-kB. Jeong et al. showed that alginic acid inhibited NF-kB activation. The authors suggested that alginic acid acts as a mast cell stabilizer and as an inhibitor of NF-kB activation, and has various regulatory effects, which might explain its anti-anaphylactic and anti-inflammatory properties [60]. We can speculate that the decreased levels of pro-inflammatory cytokines observed in our experiments are related to the inhibition of the NF-kB signal pathway; however, further experiments are necessary to test this hypothesis.

It should be pointed out that the alginate isolated from algae could be contaminated with various impurities such as heavy metals, endotoxins, proteins, and polyphenol compounds, which could increase the immunogenic response. Therefore, to assure the high purity of alginate, proper decontamination methods should be applied during the extraction procedure [61,62,63].

## 4. Materials and Methods

### 4.1. Algae Material and Chemicals

Alginate was isolated from *Cystoseira crinita* (Desf.) Bory, collected near Arapya beach, Black Sea region, Bulgaria, in July 2019. The botanical identification and algae mass pretreatment were previously described in our paper [7].

Solutions for injection of diclofenac sodium (Almiral^®^, Medochemie, Limassol, Cyprus), dexamethasone phosphate (Dexamethasone KRKA^®^, KRKA, Novo Mesto, Slovenia), and heparin sodium (Heparinum WZF^®^, Warsaw Pharmaceutical Works Polfa S.A., Warsaw, Poland) were purchased from a pharmacy store. Alginic acid sodium salt (Product No. 180947), lipopolysaccharides from *E. coli* O55:B5 (LPS), histamine, λ-carrageenan, and all other reagents were obtained from Sigma Aldrich and were of analytical grade. All tested alginates, histamine, λ-carrageenan, and LPS were dissolved in saline.

### 4.2. Animals

Male Wistar rats with an average weight of 140–280 g were used. Animals were housed under standard laboratory conditions: a 12:12 h light/dark cycle, a temperature of 22 ± 1 °C, humidity 45%, food, and water *ad libitum*.

### 4.3. Extraction of Alginate

**Figure 8:**
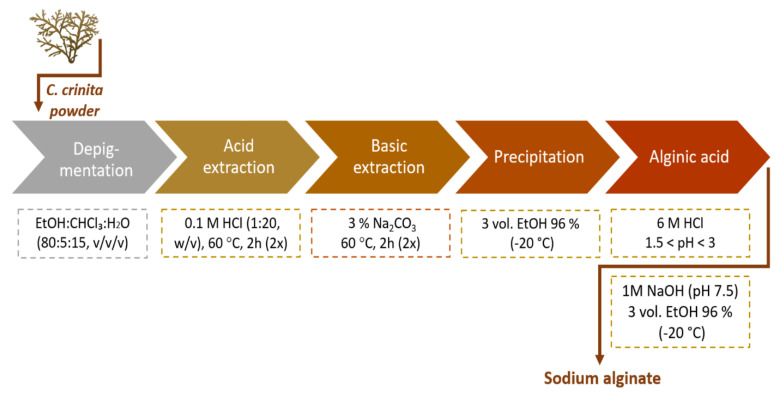
Extraction process of alginate from *C. crinita*.

*C. crinita* sodium alginate was extracted using an alkaline solvent, according to the protocol described by Hentati et al. [18] and Trica et al. [24]. Primary, 30 g *C. crinita* algae powder was soaked with an ethanol:chloroform:water solution (80:5:15, *v*/*v*/*v*) for depigmentation and delipidation. Then, the residue was washed with distilled water and added to 0.1 M HCl (600 mL, 2 h, 60 °C, 650 rpm). The acid treatment was repeated twice and then the wet pellet was recovered by centrifugation (40 min, 5000 rpm, 4 °C). The excess acid was removed by washing with distilled water. Subsequently, the alginate was extracted in 600 mL 3% Na_2_CO_3_ (pH 11) for 2 h, at a temperature of 60 °C under continuous stirring (650 rpm). The obtained extract was separated by centrifugation (40 min, 5000 rpm, 4 °C) and the filtrate was precipitated with 3 volumes of 96% ethanol (−20 °C). The precipitate was recovered by centrifugation, resolubilized in distilled water (30 g/L), and then acidified with 6 M HCl to precipitate the alginic acid at a pH value of 1.5 < pH < 3. Afterwards, the alginic acid was resuspended in distilled water (30 g/L), and the solution was neutralized to pH 7.5 using 1 M NaOH. The sodium alginate was precipitated by three volumes of 96% ethanol (−20 °C) with slow stirring, separated by centrifugation, and dried at 50 °C (Figure 8).

### 4.4. Chemical Analyses of C. crinita Alginate

The uronic acid content in *C. crinita* alginate was determined according to the methodology of Blumenkrantz and Asboe-Hansen [64]. Neutral sugars were quantified by the phenol–sulfuric acid method of Dubois et al. [65]. The sulfation degree was estimated following the turbidimetric method described by Dodgson and Price [66]. Total polyphenols were measured following the procedure of Singleton et al. [67] using Folin–Ciocalteu reagent. Protein concentration was evaluated by the Bradford method, calibrated against bovine serum albumin [68].

### 4.5. FTIR Spectroscopy

Fourier-Transform Infrared (FTIR) measurements were carried out using a Nicolet iS 10 FTIR spectrometer (Thermo Fisher Scientific, Pittsburgh, PA, USA), equipped with a diamond attenuated total reflection (ATR) accessory. The IR spectra (64 scans) were recorded at room temperature (referenced against air) with a wavenumber range of 650–4000 cm^−1^ and a resolution of 4 nm.

### 4.6. SEC-MALS

Molecular weight determination, described in our previous research [7], was performed by size-exclusion chromatography (SEC) equipped with multi-angle light scattering (MALS) (MiniDAWN TREOS II, Wyatt Technology Corporation, Santa Barbara, CA, USA) and a refractive index detector (RID-10 A, Shimadzu, Duisburg, Germany). Briefly, columns (SB-G guard column and three columns in series SB-806 HQ, SB-804 HQ, and SB-803 HQ, 300 mm L × 8 mm I.D., Shodex Showa Denko K.K., Tokyo, Japan) were eluted with NaNO_3_, 0.1 M, and NaN_3_, 2.5 mM, at a volume flow of 0.5 mL/min (LC-20AD, Shimadzu, Duisburg, Germany). Alginate (2.5 mg/mL) was filtered through a 0.45 µm membrane filter (Grace Altech, Columbia, USA) and was injected through a 100 µL full loop. Data acquisition and processing were performed using ASTRA 7.2.2 software. Specific refractive index increments (dn/dc) of 0.150 were used according to the literature.

### 4.7. ^1^H NMR Analysis

The freeze-dried samples were dissolved in D_2_O at 10–15 g/L. The ^1^H NMR spectrum was recorded at 80 °C on a Bruker Avance 500 MHz spectrometer operating at 500.08 MHz for ^1^H, using a multinuclear probe BBI 5 mm. A classical 1D proton was acquired. The sequence repeat was -D1-30°-AQ: where D1 (4 s) is the relaxation delay, 30° is the already determined 30° radio-frequency pulse length and AQ (3.27 s) is the data acquisition time. The spectrum was acquired using 8 scans of 64 K data points, using spectral widths of 10,0000 Hz. The resulting ^1^H spectrum was manually phased, baseline-corrected, and calibrated to TMSP (TriMethyl Silyl propionate) at 0 ppm, all using TopSpin 3.6 (BRUKER BioSpin, Rheinstetten, Germany).

### 4.8. Histamine-Induced Paw Edema

The experimental design was described in our previous research [7]. Forty male Wistar rats (weight 140–200 g) were divided into five groups (*n* = 8) and treated intraperitoneally as follows: 1st group (control)—treated with saline (0.1 mL/100 g bw), 2nd group (diclofenac)—treated with diclofenac sodium in a dose of 25 mg/kg bw, 3rd group (alginate standard)—treated with 100 mg/kg bw alginic acid sodium salt standard, 4th group (alginate test 25 mg/kg)—treated with 25 mg/kg bw alginate from *C. crinita*, and 5th group (alginate test 100 mg/kg)—treated with 100 mg/kg bw alginate from *C. crinita*. The volume of each injection was 100 μL/100 g bw. One hour after the treatment, the animals received a subplantar injection of 100 μL of a 0.1% solution of histamine in saline into the right hind paw. Hind paw volume was measured immediately before histamine injection and at the 5th, 15th, 30th, 60th, 90th, and 120th min with a plethysmometer (Ugo Basile, Gemonio, Italy). The paw edema was calculated according to the formula:

Percentage of increase (%) = [(*Vn* − *V_0_*)/*V_0_*] × 100 (7)

where *Vn* = the volume of the right hind paw registered after histamine injection at the n-th min; and *V_0_* = the volume of the right hind paw registered for the same animal before histamine injection.

### 4.9. Detection of Pro-Inflammatory and Anti-Inflammatory Cytokines

The experimental design was described in our previous research [7]. Twenty-four male Wistar rats (weight 200–280 g) were divided into three groups (n = 8) and treated intraperitoneally as follows: 1st group (control)—treated with saline (0.1 mL/100 g bw), 2nd group (alginate 25 mg/kg)—treated with 25 mg/kg bw alginate from *C. crinita*, and 3rd group (alginate 100 mg/kg)—treated with 100 mg/kg bw alginate from *C. crinita*. Thirty minutes after the application, a solution of LPS was injected intraperitoneally in a dose of 250 μg/kg bw. Four hours after the LPS application, the rats were sacrificed, and blood samples were collected in monovettes. The monovettes were transported immediately in an ice container to the Department of Microbiology and Immunology.

In the Department of Microbiology and Immunology, blood samples and peritoneal fluids were immediately centrifuged at 1000× *g* for 10 min at room temperature. The supernatants were subsequently achieved, aliquoted (250–500 μL) to avoid repeated freeze–thaw cycles, and stored at −80 °C until the analysis was performed. The serum concentrations of IL-1β, TNF-α, IL-6, and IL-10 and the concentrations of TNF-α in the peritoneal fluid were measured by a specific enzyme-linked immunosorbent assay (ELISA) using pre-coated strip plates. The tests were performed using the Rat IL-1β ELISA KIT of Diaclone (CEDEX—Besançon, Franche-Comté, France), Rat TNF-α ELISA KIT of Diaclone (CEDEX—Besançon, Franche-Comté, France), Rat IL-6 ELISA KIT of Diaclone (CEDEX—Besançon, Franche-Comté, France), and Rat IL-10 ELISA KIT of Diaclone (CEDEX—Besançon, Franche-Comté, France), strictly following the manufacturer’s instructions. The optical density was detected at 450 nm with an optional 620 nm reference filter using the Tecan Sunrise Microplate Reader (Tecan Austria GmbH, Groedig, Salzburg, Austria) and Magellan™ Data Analysis Software (Tecan Trading AG, V 7.2., Männedorf, Switzerland). Each sample concentration was calculated from the linear equation derived from the standard curve of the concentrations of the cytokine. The concentrations of cytokines were presented as picograms per milliliter (pg/mL).

### 4.10. Carrageenan-Induced Peritonitis

The experiment was performed as described by de Carvalho et al. [69]. Twenty-four male Wistar rats (weight 170–280 g) were divided into three groups (n = 8) and treated intraperitoneally as follows: 1st group (control)—treated with saline (0.1 mL/100 g bw), 2nd group (dexamethasone)—treated with dexamethasone phosphate in a dose of 0.2 mg/kg bw, and 3rd group (alginate)—treated with 25 mg/kg bw alginate from *C. crinita*. One hour after the application, 1 mL of λ-carrageenan solution (500 μg/mL) was injected intraperitoneally. Four hours after carrageenan application, the rats were sacrificed, and peritoneal fluid was obtained after washing the peritoneal cavity with 10 mL of saline containing 50 UI of heparin. The abdominal part of the rats was massaged gently, and a volume of 5 mL peritoneal fluid was obtained from each animal. The monovettes containing the fluid were transported immediately to the Department of Microbiology and Immunology in an ice container.

### 4.11. Statistical Analysis

Statistical analysis was performed using SPSS 17.0. The normal distribution was evaluated with the One-sample Kolmogorov–Smirnov test. One-way ANOVA and Bonferroni post hoc tests were employed for further analysis. The number of tested animals is given as n. The results are presented as mean ± SEM and are considered significant at *p* < 0.05.

## 5. Conclusions

Alginate isolated from *C. crinita* harvested in the Bulgarian Black Sea exhibited significant anti-inflammatory activity in the histamine-induced model of paw inflammation and reduced the levels of some pro-inflammatory cytokines (IL-1β, TNF-α, and IL-6) in serum after LPS application in rats. Alginate did not significantly change the level of anti-inflammatory cytokine IL-10. The anti-phlogistic activity of alginate is probably related to a decreased secretion of pro-inflammatory cytokines. However, further studies are required to elucidate the key molecular mechanism involved in the anti-inflammatory potential of alginate isolated from *C. crinita*.

## Figures and Tables

**Table 1:** Extraction yield and chemical composition of *C. crinita* alginate.

Sample	ExtractionYield (%)	Neutral Sugars (%*, w/w*)	Uronic Acid (%*, w/w*)	Sulfates (%*, w/w*)	Total Polyphenols (%)	Protein (%)
*C. crinita* alginate	20.18 ± 1.72	19.66 ± 1.05	50.14 ± 1.12	0.63 ± 0.02	<0.10	<0.04

**Table 2:** Structural characterization of alginate extracted from *C. crinita* harvested in the Bulgarian Black Sea.

Fraction	F_G_	F_M_	F_GG_	F_GM_ or F_MG_	F_MM_	M/G
Alginate	0.495	0.505	0.280	0.216	0.289	1.018

**Table 3:** Mean percentages of increase in the rat paw volume in a model of histamine-induced edema after treatment with saline (controls), diclofenac sodium in a dose of 25 mg/kg bw, alginic acid sodium salt standard in a dose of 100 mg/kg bw, and alginate test from *C. crinita* in doses of 25 and 100 mg/kg bw, respectively.

	Time Point	5th Minute	15th Minute	30th Minute	60th Minute	90th Minute	120th Minute
Groups							
Controls	Mean (%)	31.94	50.72	59.94	52.32	43.44	38.58
	SEM	2.23	4.05	3.85	2.98	4.52	5.03
diclofenac 25 mg/kg	Mean (%)	12.57 **	19.25 ***	20.48 ***	18.05 ***	15.24 ***	12.90 ***
	SEM	1.73	1.50	2.13	1.91	2.40	2.22
alginate standard 100 mg/kg	Mean (%)	19.77	28.73 **	27.72 ***	18.19 ***	15.53 ***	15.23 ***
	SEM	2.09	4.26	3.34	2.79	3.01	3.46
alginate test 25 mg/kg	Mean (%)	17.39 *	21.23 ***	24.64 ***	10.73 ***	7.04 ***	6.10 ***
	SEM	4.68	3.57	3.43	1.70	1.47	1.85
alginate test 100 mg/kg	Mean (%)	21.31	28.99 **	25.46 ***	12.16 ***	10.37 ***	8.27 ***
	SEM	4.49	3.75	5.46	2.83	3.06	2.53

* *p* < 0.05 vs. controls at the same time; ** *p* < 0.01 vs. controls at the same time; *** *p* < 0.001 vs. controls at the same time.

## Data Availability

The data presented in this study are available on request from the corresponding author.

## References

[1] Mišurcová L., Škrovánková S., Samek D., Ambrožová J., Machů L. (2012). Health benefits of algal polysaccharides in human nutrition. Adv. Food Nutr. Res..

[2] Holdt S.L., Kraan S. (2011). Bioactive compounds in seaweed: Functional food applications and legislation. J. Appl. Phycol..

[3] Ye H., Wang K., Zhou C., Liu J., Zeng X. (2008). Purification, antitumor and antioxidant activities in vitro of polysaccharides from the brown seaweed *Sargassum pallidum*. Food Chem..

[4] De Jesus Raposo M.F., De Morais A.M.B., De Morais R.M.S.C. (2015). Marine polysaccharides from algae with potential biomedical applications. Mar. Drugs.

[5] Okolie C.L., CK Rajendran S.R., Udenigwe C.C., Aryee A.N., Mason B. (2017). Prospects of brown seaweed polysaccharides (BSP) as prebiotics and potential immunomodulators. J. Food Biochem..

[6] Lim S.J., Aida W.M.W., Venkatesan J., Anil S., Kim S.K. (2017). Extraction of Sulfated Polysaccharides (Fucoidan) From Brown Seaweed. Seaweed Polysaccharides.

[7] Apostolova E., Lukova P., Baldzhieva A., Delattre C., Molinié R., Petit E., Elboutachfaiti R., Nikolova M., Iliev I., Murdjeva M. (2022). Structural characterization and in vivo anti-inflammatory activity of fucoidan from *Cystoseira crinita* (Desf.) Borry. Mar. Drugs.

[8] Kadam S.U., Tiwari B.K., O’donnell C.P. (2015). Extraction, structure and biofunctional activities of laminarin from brown algae. Int. J. Food Sci. Technol..

[9] Déléris P., Nazih H., Bard J.M., Fleurence J., Levine I. (2016). Seaweeds in human health. Seaweed in Health and Disease Prevention.

[10] Priyan Shanura Fernando I., Kim K.N., Kim D., Jeon Y.J. (2019). Algal polysaccharides: Potential bioactive substances for cosmeceutical applications. Crit. Rev. Biotechnol..

[11] Rioux L.-E., Turgeon S.L., Beaulieu M. (2007). Characterization of polysaccharides extracted from brown seaweeds. Carbohydr. Polym..

[12] Lee K.Y., Mooney D.J. (2012). Alginate: Properties and biomedical applications. Prog. Polym. Sci..

[13] Sun J.C., Tan H.P. (2013). Alginate-based biomaterials for regenerative medicine applications. Materials.

[14] Xing M., Cao Q., Wang Y., Xiao H., Zhao J., Zhang Q., Ji A., Song S. (2020). Advances in research on the bioactivity of alginate oligosaccharides. Mar. Drugs..

[15] Szekalska M., Puciłowska A., Szymańska E., Ciosek P., Winnicka K. (2016). Alginate: Current use and future perspectives in pharmaceutical and biomedical applications. Int. J. Polym. Sci..

[16] Milkova T., Talev G., Christov R., Dimitrova-Konaklieva S., Popov S. (1997). Sterols and volatiles in *Cystoseira barbata* and *Cystoseira crinita* from the black sea. Phytochemistry.

[17] Sellimi S., Younes I., Ayed H.B., Maalej H., Montero V., Rinaudo M., Dahia M., Mechichi T., Hajji M., Nasri M. (2015). Structural, physicochemical and antioxidant properties of sodium alginate isolated from a Tunisian brown seaweed. Int. J. Biol. Macromol..

[18] Hentati F., Delattre C., Ursu A.V., Desbrières J., Le Cerf D., Gardarin C., Abdelkafi S., Michaud P., Pierre G. (2018). Structural characterization and antioxidant activity of water-soluble polysaccharides from the Tunisian brown seaweed *Cystoseira compressa*. Carbohydr. Polym..

[19] Benslima A., Sellimi S., Hamdi M., Nasri R., Jridi M., Cot D., Li S., Nasri M., Zouari N. (2021). The brown seaweed *Cystoseira schiffneri* as a source of sodium alginate: Chemical and structural characterization, and antioxidant activities. Food Biosci..

[20] Pereira L., Sousa A., Coelho H., Amado A.M., Ribeiro-Claro P.J. (2003). Use of FTIR, FT-Raman and 13C-NMR spectroscopy for identification of some seaweed phycocolloids. Biomed. Eng..

[21] Kaidi S., Bentiss F., Jama C., Khaya K., Belattmania Z., Reani A., Sabour B. (2022). Isolation and structural characterization of alginates from the Kelp species *Laminaria ochroleuca* and *Saccorhiza polyschides* from the Atlantic Coast of Morocco. Colloids Interfaces.

[22] Aarstad O.A., Tøndervik A., Sletta H., Skjåk-Bræk G. (2012). Alginate sequencing: An analysis of block distribution in alginates using specific alginate degrading enzymes. Biomacromolecules.

[23] Fenoradosoa T.A., Ali G., Delattre C., Laroche C., Petit E., Wadouachi A., Michaud P. (2010). Extraction and characterization of an alginate from the brown seaweed *Sargassum turbinarioides* Grunow. J. Appl. Phycol..

[24] Trica B., Delattre C., Gros F., Ursu A.V., Dobre T., Djelveh G., Michaud P., Oancea F. (2019). Extraction and characterization of alginate from an edible brown seaweed (*Cystoseira barbata*) harvested in the Romanian Black Sea. Mar. Drugs.

[25] Grasdalen H., Larsen B., Smidsrød O. (1979). A pmr study of the composition and sequence of uronate residues in alginates. Carbohydr. Res..

[26] Heyraud A., Gey C., Leonard C., Rochas C., Girond S., Kloareg B. (1996). NMR spectroscopy analysis of oligoguluronates and oligomannuronates prepared by acid or enzymatic hydrolysis of homopolymeric blocks of alginic acid. Application to the determination of the substrate specificity of Haliotis tuberculata alginate lyase. Carbohydr. Res..

[27] Davis T.A., Ramirez M., Mucci A., Larsen B. (2004). Extraction, isolation and cadmium binding of alginate from *Sargassum* spp.. J. Appl. Psychol..

[28] Rhein-Knudsen N., Ale M.T., Ajalloueian F., Meyer A.S. (2017). Characterization of alginates from Ghanaian brown seaweeds: *Sargassum* spp. and *Padina* spp.. Food Hydrocoll..

[29] Rashedy S.H., Abd El Hafez M.S., Dar M.A., Cotas J., Pereira L. (2021). Evaluation and characterization of alginate extracted from brown seaweed collected in the Red Sea. Appl. Sci..

[30] Permatasari A.A.A.P., Rosiana I.W., Wiradana P.A., Lestari M.D., Widiastuti N.K., Kurniawan S.B., Widhiantara I.G. (2022). Extraction and characterization of sodium alginate from three brown algae collected from Sanur Coastal Waters, Bali as biopolymer agent. Biodiversitas J. Biol. Divers..

[31] Kelly B.J., Brown M.T. (2000). Variations in the alginate content and composition of Durvillaea antarctica and D. willana from southern New Zealand. J. Appl. Phycol..

[32] Bouissil S., El Alaoui-Talibi Z., Pierre G., Michaud P., El Modafar C., Delattre C. (2020). Use of alginate extracted from Moroccan brown algae to stimulate natural defense in date palm roots. Molecules.

[33] Saji S., Hebden A., Goswami P., Du C. (2022). A brief review on the development of alginate extraction process and its sustainability. Sustainability.

[34] Aitouguinane M., Alaoui-Talibi Z.E., Rchid H., Fendri I., Abdelkafi S., El-Hadj M.D.O., Boual Z., Dubessay P., Michaud P., Traïkia M. (2022). Polysaccharides from Moroccan green and brown seaweed and their derivatives stimulate natural defenses in olive tree leaves. Appl. Sci..

[35] Hachemi-Benmalek N., Nouani A., Benchabane A. (2019). Valorization of brown algae (*Cystoseira caespitosa*) from local region in Algeria for sodium alginate extraction and their application in the immobilization of microbial pectinases. ALJEST.

[36] Day D.F., Kaplan D.L. (1998). Alginates. Biopolymers from Renewable Resources.

[37] Gacesa P. (1988). Alginates. Carbohydr. Polym..

[38] Abka-Khajouei R., Tounsi L., Shahabi N., Patel A.K., Abdelkafi S., Michaud P. (2022). Structures, properties and applications of alginates. Mar. Drugs.

[39] Hadj Ammar H., Lajili S., Ben Said R., Le Cerf D., Bouraoui A., Majdoub H. (2015). Physico-chemical characterization and pharmacological evaluation of sulfated polysaccharides from three species of Mediterranean brown algae of the genus Cystoseira. Daru J. Fac. Pharm. Tehran Univ. Med. Sci..

[40] Siebers U., Horcher A., Bretzel R.G., Federlin K., Zekorn T. (1997). Alginate-based microcapsules for immunoprotected islet transplantation. Ann. N. Y. Acad. Sci..

[41] Kammerlander G., Eberlein T. (2003). An assessment of the wound healing properties of Algisite M dressings. Nurs. Times.

[42] Ohsumi H., Hirata H., Nagakura T., Tsujii M., Sugimoto T., Miyamoto K., Horiuchi T., Nagao M., Nakashima T., Uchida A. (2005). Enhancement of perineurial repair and inhibition of nerve adhesion by viscous injectable pure alginate sol. Plast. Reconstr. Surg..

[43] Hori Y., Winans A.M., Huang C.C., Horrigan E.M., Irvine D.J. (2008). Injectable dendritic cell-carrying alginate gels for immunization and immunotherapy. Biomaterials.

[44] Abramowitz L., Weyandt G.H., Havlickova B., Matsuda Y., Didelot J.M., Rothhaar A., Sobrado C., Szabadi A., Vitalyos T., Wiesel P. (2010). The diagnosis and management of haemorrhoidal disease from a global perspective. Aliment. Pharmacol. Ttherap..

[45] Slezka I.E., Miroshichenko V.A., Vostrikova O.G., Ziganshina O.A. (1998). Applications of bioactive compounds from marine organisms in atherosclerosis prophylaxis in children, new biomedical technologies using bioactive additives. Proceedings of the All Russian Conference.

[46] Hasegawa T., Takahashi T., Inada Y., Yamada C., Tanaka Y. (1989). Reparative effects of sodium alginate (Alloid G) on radiation stomatitis. Nihon Igaku Hoshasen Gakkai Zasshi. Nippon Acta Radiol..

[47] Katayama S., Ohshita J., Sugaya K., Hirano M., Momose Y., Yamamura S. (1998). New medicinal treatment for severe gingivostomatitis. Int. J. Mol. Med..

[48] Yang D., Jones K.S. (2009). Effect of alginate on innate immune activation of macrophages. J. Biomed. Mater. Res. A.

[49] Son E.H., Moon E.Y., Rhee D.K., Pyo S. (2001). Stimulation of various functions in murine peritoneal macrophages by high mannuronic acid-containing alginate (HMA) exposure in vivo. Int. Immunopharmacol..

[50] Otterlei M., Ostgaard K., Skjåk-Braek G., Smidsrød O., Soon-Shiong P., Espevik T. (1991). Induction of cytokine production from human monocytes stimulated with alginate. J. Immunother..

[51] Takahashi K., Watanuki Y., Yamazaki M., Abe S. (1988). Local induction of a cytotoxic factor in a murine tumor by systemic administration of an antitumor polysaccharide, MGA. Br. J. Cancer.

[52] Mirshafiey A., Rehm B.H., Rehm B.H. (2009). Alginate and its comonomer mannuronic acid: Medical relevance as drugs. Alginates: Biology and Applications.

[53] Mirshafiey A., Khodadadi A., Rehm B.H., Khorramizadeh M.R., Eslami M.B., Razavi A., Saadat F. (2005). Sodium alginate as a novel therapeutic option in experimental colitis. Scand. J. Immunol..

[54] Razavi A., Khodadadi A., Eslami M.B., Eshraghi S., Mirshafiey A. (2008). Therapeutic effect of sodium alginate in experimental chronic ulcerative colitis. Iran J. Allergy Asthma Immunol..

[55] Mirshafiey A., Borzooy Z., Abhari R.S., Razavi A., Tavangar M., Rehm B.H. (2005). Treatment of experimental immune complex glomerulonephritis by sodium alginate. Vascul. Pharmacol..

[56] De la Coba F., Aguilera J., Figueroa F.L., De Gálvez M.V., Herrera E. (2009). Antioxidant activity of mycosporine-like amino acids isolated from three red macroalgae and one marine lichen. J. Appl. Phycol..

[57] Sarithakumari C.H., Renju G.L., Kurup G.M. (2013). Anti-inflammatory and antioxidant potential of alginic acid isolated from the marine algae, *Sargassum wightii* on adjuvant-induced arthritic rats. Inflammopharmacology.

[58] Mo S.J., Son E.W., Rhee D.K., Pyo S. (2003). Modulation of tnf-α-induced icam-1 expression, no and h 2 0 2 production by alginate, allicin and ascorbic acid in human endothelial cells. Arch. Pharm. Res..

[59] Asada M., Sugie M., Inoue M., Nakagomi K., Hongo S., Murata K., Irie S., Takeuchi T., Tomizuka N., Oka S. (1997). Inhibitory effect of alginic acids on hyaluronidase and on histamine release from mast cells. Biosci. Biotechnol. Biochem..

[60] Jeong H.J., Lee S.A., Moon P.D., Na H.J., Park R.K., Um J.Y., Kim H.M., Hong S.H. (2006). Alginic acid has anti-anaphylactic effects and inhibits inflammatory cytokine expression via suppression of nuclear factor-κB activation. Clin. Exp. Allergy.

[61] Ménard M., Dusseault J., Langlois G., Baille W.E., Tam S.K., Yahia L.H., Zhu X.X., Hallé J.P. (2010). Role of protein contaminants in the immunogenicity of alginates. J. Biomed. Mater..

[62] Greco M., Saez C.A., Brown M.T., Bitonti M.B. (2014). A simple and effective method for high quality co-extraction of genomic DNA and total RNA from low biomass *Ectocarpus siliculosus*, the model brown alga. PLoS ONE.

[63] Skjak-Brek G., Martinsen A., Guiry L., Bluden G. (1991). Applications of some algal polysaccharides in biotechnology. Seaweed Resources in Europe: Uses and Potentials.

[64] Blumenkrantz N., Asboe-Hansen G. (1973). New method for quantitative determination of uronic acids. Anal. Biochem..

[65] Dubois M., Gilles K., Hamilton J.K., Rebers P.A., Smith F. (1951). A colorimetric method for the determination of sugars. Nature.

[66] Dodgson K.S., Price R.G. (1962). A note on the determination of the ester sulphate content of sulphated polysaccharides. Biochem. J..

[67] Singleton V.L., Orthofer R., Lamuela-Raventós R.M. (1999). Analysis of total phenols and other oxidation substrates and antioxidants by means of Folin-Ciocalteu reagent. Method. Enzymol..

[68] Bradford M.M. (1976). A rapid and sensitive method for the quantitation of microgram quantities of protein utilizing the principle of protein-dye binding. Anal. Biochem..

[69] de Carvalho A.M., Rocha N.F., Vasconcelos L.F., Rios E.R., Dias M.L., Silva M.I., de França Fonteles M.M., Filho J.M., Gutierrez S.J., de Sousa F.C. (2013). Evaluation of the anti-inflammatory activity of riparin II (O-methil-N-2-hidroxi-benzoyl tyramine) in animal models. Chem. Biol. Interact..

